# Developmental Dysplasia of the Hip in Infants: Prevalence and Risk Factors

**DOI:** 10.3390/medicina62071215

**Published:** 2026-06-23

**Authors:** Marcelo Ortega-Silva, Pablo Navarro-Cáceres, Mariano del Sol

**Affiliations:** 1 Programa de Doctorado en Ciencias Morfológicas, Facultad de Medicina, Universidad de La Frontera, Temuco 4780000, Chile; 2 Escuela de Tecnología Médica, Facultad de Ciencias, Universidad San Sebastián, Lago Panguipulli 1390, Puerto Montt 5501842, Chile; 3 Centro de Investigación en Ciencias Odontológicas (CICO), Facultad de Odontología, Universidad de La Frontera, Temuco 4811230, Chile; pablo.navarro@ufrontera.cl; 4 Facultad de Ciencias de la Salud, Universidad Autónoma de Chile, Temuco 4780000, Chile; 5 Centro de Excelencia en Estudios Morfológicos y Quirúrgicos (CEMyQ), Facultad de Medicina, Universidad de La Frontera, Temuco 4780000, Chile

**Keywords:** developmental dysplasia of the hip, prevalence, radiography, risk factors

## Abstract

*Background and Objectives*: Developmental dysplasia of the hip (DDH) is an orthopedic condition in the pediatric population, affecting between 0.1% and 3% of infants. Chile has one of the highest incidences in South America, reaching 1 per 500 live births. Given the potential of adverse consequences of DDH on infant health, preliminary studies are needed to determine its prevalence in the population and assess its association with relevant risk factors. *Materials and Methods*: The study is single-center, conducted in a Chilean population. The sample size calculation determined the use of 100 pelvic radiographs, considering a 95% confidence level and a proportion of 0.5. The infants were between 90 and 150 days old. Information was collected on possible DDH-related risk factors. For the analysis, normality tests, Chi-square tests, independent samples t-tests, Mann–Whitney U tests, and multivariate analyses were applied. *Results*: The prevalence of DDH was determined to be 12%, affecting the left hip to a greater extent. Female infants had a higher frequency of DDH. A statistically significant association was found between the prevalence of DDH and the presence of asymmetry in the abduction of the hip joint (*p* = 0.023), acetabular roof obliquity (*p* = 0.003), left hip involvement (*p* = 0.002), and height at two months of age (*p* = 0.016). *Conclusions*: The prevalence of DDH in infants was higher than that reported in the literature. However, with regard to sex, the data coincide with those previously reported by other authors.

## 1. Introduction

Developmental dysplasia of the hip (DDH) is one of the most common orthopedic conditions in the pediatric population [1], affecting between 0.1% and 3% of the population [2]. This condition was considered congenital, present from birth. However, it was found that many cases presented late or were not diagnosed until long after birth; therefore, the term “developmental” was adopted to reflect that the condition can arise during the uterine period or in the postnatal period, as the infant’s hips develop [3]. This term is more inclusive than the previous “congenital hip dislocation,” as it includes other anomalies besides dislocation [4]. It is now known to be a dynamic condition of an individual’s development, which can deteriorate or improve over time [5,6].

Typically, the femoral head and acetabulum fit together anatomically in a stable and congruent ball-and-socket joint [7]. DDH includes a broad spectrum of abnormal hip development during early childhood. It involves an alteration in the relationship between the acetabulum and the proximal femur, which may be due to inadequate development of the acetabulum, the femoral head, or both, affecting the laxity of the surrounding ligaments [8].

Infants with untreated dislocation may present with discrepancies in the length of the lower limbs or deformities and pain in the knee joint, secondary scoliosis, and back pain [3]. Asymmetry in skin folds in the inguinal and gluteal regions, limitation or asymmetry in hip abduction, and gait disturbance have also been reported [6]. In some infants, DDH may not be detected until lameness becomes apparent, which is commonly identified when they begin to walk [9].

These DDH disorders must be detected early; otherwise, they can range from mild capsular laxity to early osteoarthritis, secondary damage to the femur, or movement issues [3]. This pathology can lead to substantial healthcare costs and disability in patients, with a deleterious impact on their social environment [10]. DDH is the cause of 9 to 30% of primary hip replacements and 29% in patients under 60 years of age [11,12,13].

Authors have reported a wide range of incidence and prevalence data for DDH. These data vary mainly according to geographical location and ethnic origin. This could be explained by epigenetic factors, consanguinity rates, limitations inherent to the types of studies conducted, the patient’s age at the time of testing, the diagnostic criteria used, and the sample size [1,14]. The population with the lowest incidence of DDH is African, at 0.1%, and the highest incidence is among Native Americans, at 7.6% [11].

Between 1% and 2% of newborns have subluxated or dislocated hips at birth. Sixty percent of them stabilize during the first week of life, and 88% by two months of age. Females are most affected by DDH, with a female-to-male ratio of 6:1 [15]. Despite the studies, screenings, and programs available to identify dysplasia, late diagnoses have been reported in 2.2 per 1000 infants [16]. On the other hand, 1 in 5000 cases of dysplasia are detected after 18 months of age [17]. Dislocations in DDH occur in 11.5 per 1000 infants. Likewise, an incidence of 3 to 4 dislocations per 1000 live births has been reported [18].

### Risk Factors Associated with Developmental Dysplasia of the Hip

The etiology of DDH is not yet fully understood, although certain risk factors have been consistently identified in the specialized literature. These risk factors are estimated to be present in 10–27% of affected patients; however, the mechanisms linking them to the onset of DDH remain unclear [19].

A family history of DDH increases the likelihood of developing DDH by 12 to 15.9 times [20,21]. Vaginal delivery increases the risk of a DDH diagnosis by 2.7 times compared to cesarean deliveries [21]. On the other hand, underlying pathologies or the presence of conditions such as Down syndrome, clubfoot, torticollis, adducted metatarsus, or tendon laxity increase the risk of developing DDH [22]. It has also been noted that prolonged gestation beyond 38 weeks increases the probability of DDH by 1.7 times [21]. Limited fetal mobility, caused by factors such as oligohydramnios and high birth weight, is associated with an increased risk of DDH [23]. In some populations, such as the Japanese, the use of diapers has also been reported to correlate with higher rates of DDH, attributed to the use of tight-fitting diapering practices [24]. Finally, a recent study in Tibet showed an increase in the incidence of DDH related to altitude [25].

There is also mixed evidence in the literature regarding other risk factors such as high birth weight, prematurity, multiple pregnancy, fetal packaging disorders, environmental factors, prenatal positioning, maternal age, and number of previous births [4,6,7,9,16,26].

Chile, among Latin American countries, presents one of the highest incidences of DDH in the region, reaching 1 case per 500 live births for subluxation and dislocation forms of the hip. Currently, the incidence of all forms of dysplasia has reached 5 per 100 live births [15]. In Chile, due to public health policies included in the Explicit Health Guarantees (GES), the medical team must request an anteroposterior pelvic X-ray for every patient at 3 months of age. This is used as a screening method to detect DDH, regardless of the possible risk factors that patients may have. The X-ray is analyzed by a radiologist or pediatric orthopedist, who determines the acetabular angle, which should be less than 30° [2]. This screening facilitates the early detection of DDH, enabling timely intervention and avoiding the risk of long-term issues.

Considering both global and local background information, updated data on the prevalence of DDH in the Chilean population are needed, along with an evaluation of its association with relevant risk factors. This is important due to the negative impacts on infant health, healthcare costs, and long-term complications resulting from a late diagnosis. This study seeks to contribute relevant clinical information on a condition that significantly affects the pediatric population.

## 2. Materials and Methods

### 2.1. Study Type and Sample

The methodology consisted of a descriptive observational study. The sample size for a finite population was calculated using a 95% confidence level, an expected proportion of 0.5, and a margin of error of 0.05, resulting in a required sample of 109 observation units. Subsequently, a sampling adjustment was applied, which yielded an expected sample of n = 100 observation units. In this way, 100 pediatric pelvic radiographs were analyzed together with their radiology reports.

The sample was chosen for convenience and included infants who met the following criteria: no prior diagnosis of DDH, their first screening X-ray through the GES program or privately, and aged 90–150 days. The infants in this sample were from the municipality of Temuco, Chile. In addition, to gather information on possible risk factors associated with DDH described in the literature and others of clinical interest for the present study, a survey was administered to parents/guardians based on the patient record that all infants in Chile have. The data collected included: type of feeding, age, sex, ethnicity, type of delivery, family history of DDH, uneven gluteal folds, limbs of unequal size, thigh and hip circumference, weeks of gestation, number of children, breech presentation, weight, height, and acetabular angle.

### 2.2. Radiographic Technique

X-rays were obtained based on the protocol established in the MINSAL Clinical Guide to Hip Dysplasia [2], which specifies the following parameters: the patient must be positioned supine, with the lower limbs extended, parallel, with slight traction, symmetrical, and with the knees at the zenith (without internal rotation). The X-ray beam is centered at a standard distance of 100 cm.

### 2.3. Radiography Inclusion Criteria

The X-rays included in the study had to meet the following criteria: infant age between 90 and 150 days, symmetrical hips, no rotation, well centered, no anteversion or retroversion, iliac fossae and obturator foramen of similar width, symmetrical proximal femoral epiphyses with visualization of the lesser trochanters [2], completely visible anatomical structure, no distortion of the anatomy, and adequate radiological contrast.

### 2.4. Radiological Report

Information regarding the diagnosis of dysplasia was retrieved from the radiology report. This report was issued by radiologists, who interpreted the X-ray taken in the project, providing a clinically valid report. The data reported were used to determine the prevalence of DDH. Furthermore, the radiological report includes the measurement of the acetabular angle; when this value exceeds 30°, it is indicative of DDH. On our part, we performed independent measurements of the acetabular angle in order to compare them with those reported in the radiological reports, which were consistent with the findings provided by the radiologist.

### 2.5. Statistical Analysis

The data collected were recorded in a Microsoft Excel spreadsheet. A descriptive data analysis was performed to determine the mean, standard deviation, and frequency distribution table. The Kolmogorov–Smirnov normality test, Shapiro–Wilk normality test, Pearson’s chi-square test, independent-samples t-test, Mann–Whitney U test, and multivariate analysis using binary logistic regression were applied. The data were analyzed using the statistical program SPSS Statistics for Windows (version 23.0, IBM Corporation, Armonk, NY, USA), while the Mann–Whitney U test and multivariate analysis used binary logistic regression. A *p*-value < 0.05 was chosen as the threshold for significance.

## 3. Results

Among the 100 pediatric pelvis reports used, the prevalence of DDH in the sample was 12%, of which 66.7% were female.

The left hip was the most affected, with 41.7%, followed by the right hip (25%) and bilateral involvement (33.3%).

Concerning acetabular roof obliquity, 97% of the sample had a normal conformation, while 3% showed increased obliquity.

Findings related to the development of the acetabular rims are presented in Table 1.

With regard to the type of delivery, 36% of infants were born vaginally, while 64% were born by cesarean section. An analysis of the relationship with the presence of DDH revealed that 6 cases occurred in vaginal deliveries and another 6 in cesarean deliveries.

Regarding belonging to an indigenous people, 44% of parents/guardians stated that they belonged to the Mapuche people.

In relation to prematurity, only 2 cases of premature birth were reported. Forty-one percent of the sample corresponded to firstborn infants.

Regarding feeding type, 52% of infants were exclusively breastfed (EBF). Meanwhile, 15% were fed exclusively with formula, and 33% received mixed feeding, combining EBF and formula.

In the analysis of the lower limbs, it was observed that 5% of infants had unequal lower limb length, 18% showed asymmetry in the abduction of the hip joint, and 9% had uneven gluteal folds.

Regarding fetal presentation at the time of delivery, 10% indicated a breech position, 13% did not provide this information, and 77% indicated that the position was cephalic, meaning that the head was oriented toward the birth canal.

When asked about family history of DDH, 31% indicated that a paternal or maternal relative had had this condition.

The descriptive statistics data corresponding to the scalar variables are presented in Table 2.

The intraclass correlation coefficient (ICC) was determined to assess the measurements of the acetabular angles of both the right and left hips and the thigh circumference measurements. For the acetabular angles, an ICC of 0.868 was obtained, indicating a good level of inter-rater agreement. On the other hand, for thigh circumference measurements, an ICC of 0.953 was obtained, which indicates a very good level of agreement.

In the statistical analysis of qualitative variables, no statistically significant differences were found when relating the prevalence of DDH to variables such as sex (*p* = 0.108), age (*p* = 0.685), type of delivery (*p* = 0.281), breech presentation (*p* = 0.419), ethnicity (*p* = 0.862), prematurity (*p* = 0.598), firstborn (*p* = 0.565), EBF (*p* = 0.640), formula feeding (*p* = 0.640), mixed feeding (*p* = 0.496), family history of DDH (*p* = 0.632), lower limb asymmetry (*p* = 0.397), gluteal folds (*p* = 0.931), and development of the cotyloid eyebrows (*p* = 0.107). However, a statistically significant association was found between the prevalence of DDH and the presence of asymmetry in hip abduction (*p* = 0.023), acetabular roof obliquity (*p* = 0.003), and the left hip (*p* = 0.002).

No statistically significant differences were found between the prevalence of DDH and variables such as right thigh circumference (*p* = 0.388) and left thigh circumference (*p* = 0.432), pelvic circumference (*p* = 0.542), birth weight (*p* = 0.264), weight at one month of age (*p* = 0.304), weight at two months of age (*p* = 0.312), weight at three months of age (*p* = 0.883), and weight at the time of examination (*p* = 0.299), birth length (*p* = 0.086), height at one month of age (*p* = 0.182), height at three months of age (*p* = 0.346), height at the time of examination (*p* = 0.174), number of children (*p* = 0.706), and weeks of gestation (*p* = 0.179). However, a statistically significant difference was observed when relating the prevalence of DDH to the right (*p* = 0.001) and left (*p* = 0.001) acetabular angles and height at two months of age (*p* = 0.016).

### Binary Logistic Regression

A binary logistic regression (BLR) model was constructed considering the presence of dysplasia (1 = Yes, 0 = No) as the response variable. The predictor variables included in the model were: Abduction Asymmetry (*p* = 0.023), acetabular roof obliquity (*p* = 0.003), and Affected Hip (*p* = 0.002), all of which were statistically significant, indicating that their association with the presence of dysplasia is different from zero at the conventional level of significance. The BLR model had an accuracy rate of 88%, indicating adequate predictive power to classify the presence or absence of dysplasia correctly.

Nagelkerke’s statistic (R^2^ = 0.988) explained 98.8% of the variability of the variable presence of dysplasia, which is explained by the heterogeneity of the variables Abduction Asymmetry, Obliquity Roof, and Affected Hip.

## 4. Discussion

This study documents relevant information on a highly prevalent condition in the Chilean pediatric population, the early detection of which is essential due to the magnitude of complications it can cause in infants. Notably, DDH is included in the Explicit Health Guarantees (GES) in Chile, which ensures universal access to radiological screening and, if the diagnosis is confirmed, to timely treatment.

Based on the analysis of radiological reports, a prevalence of DDH of 12% was determined in the sample, a value considerably higher than that reported in the literature, where prevalence ranges between 0.1% and 7% [2,11,27,28]. As previously described, the prevalence of DDH varies widely, depending on factors such as geographic region, ethnic origin, and epigenetic conditions [1]. This prevalence estimate should be interpreted with caution, as it represents a preliminary result within the data that remain to be collected at the national level.

In the region, Brazilian authors report a DDH prevalence of 5.46%, based on ultrasound screening in newborns [29]. Another Brazilian study reports a prevalence ranging from 0.75 to 56.4 cases per 1000 infants [30]. A Colombian study reports an incidence of DDH with late dislocation ranging from 4.3 to 8.3 per 1000 live births, considering children older than 3 months [31]. Native American populations show a prevalence of 7.6% [11]. No clear information was identified for other countries such as Argentina, Peru, and Bolivia, among others. It is particularly noteworthy that the prevalence observed in Chile is higher than that reported in neighboring regions. However, it is important to consider that this value corresponds to a specific sample of the Chilean population and cannot be directly extrapolated to other regions of the country; we consider this a limitation of the study. Given Chile’s extensive geographic distribution and the diversity of its cultural admixture, regional variations in prevalence are to be expected. In this context, the results should be considered exploratory and a starting point for addressing this issue at the local level. Multicenter studies are therefore required to estimate a nationally representative prevalence.

The prevalence of DDH in the left hip was 41.7%, higher than that observed in the right hip and in bilateral presentations. This finding is consistent with the literature, which describes a 3:1 ratio in favor of the left side [32], with approximately 60% of cases of unilateral left DDH and 20% of bilateral cases [15]. In relation to bilaterality, 33.3% was reported in this sample, a relevant value as it is higher than that described in other studies. This finding could be related to additional factors, such as the absence of the ossification nucleus of the femoral head, a condition that is often associated with the presence of DDH. Likewise, a statistically significant association was found between the presence of DDH and the involvement of the left hip. One possible explanation for this predominance is related to the most frequent fetal position in the uterus, described as occipito-left-anterior. In this position, the fetal head is oriented caudally, the back toward the maternal abdomen, and the head slightly anterior, which places the left hip closer to the maternal spine. This situation could generate greater pressure or restriction of movement of the developing hip, favoring the appearance of DDH on that side [32].

With regard to gender, a higher prevalence of DDH was observed in females, at 6.7%, consistent with reports in the literature [32]. However, this value is higher than that described by other authors, who report prevalences of 1.46% in women and 0.66% in men [28]. Several studies have indicated that female infants are between 4 and 9 times more likely to develop DDH [15,16,19,21]. This greater susceptibility has been attributed to the action of maternal hormones, such as estrogens, progestogens, and relaxin, which promote relaxation of the joint capsule and ligaments [32,33]. Based on this preliminary result, female infants should continue to be clinically regarded as a higher-risk group, particularly in terms of early detection, in order to prevent more severe complications in the future.

With regard to the obliquity of the acetabular roof, it was observed that 97% of cases had normal morphology, which is a relevant finding in radiographic analysis, as it reflects adequate seating of the femoral head in the acetabulum and indirectly suggests a stable joint [34]. The acetabular concavity develops progressively as a result of the pressure exerted by the femoral head on the acetabular roof during growth [35]. Considering that the femoral ossification nucleus is in a cartilaginous state and not completely visible on the radiograph, these findings enable an indirect analysis of the relationship between the two structures. Considering that the femoral ossification nucleus remains cartilaginous and is not fully visible on radiography, these findings allow for indirect assessment of the relationship between these structures. This, in turn, facilitates a more robust morphological, radiological, and diagnostic evaluation through the use of radiography in the assessment of DDH.

The development of the acetabular rim corresponds to the degree of ossification of the superolateral edge of the acetabulum; when this is scarce or absent, it suggests incomplete ossification or dysplastic acetabular development. Together with the obliquity of the acetabular roof and the acetabular angle, this variable contributes to a more accurate diagnosis [34]. In this sample, 62% presented adequate development of this structure, suggesting correct pelvic bone development. When correlating this variable with the presence of DDH, it was observed that 41.7% of patients with dysplasia had poor development of the superolateral rim of the acetabulum. However, no statistical significance was found between these variables. Nevertheless, from a descriptive statistical point of view, this variable shows a high proportion in cases with DDH, so its inclusion in future studies with larger samples is considered relevant.

Regarding the type of delivery, cesarean section predominated with 64%, while vaginal delivery accounted for 36%. When analyzing this variable in subjects diagnosed with DDH, it was observed that, of the 12 cases identified, 6 were born by vaginal delivery and 6 by cesarean section, with no significant differences. The literature has indicated that vaginal delivery could be associated with a higher risk of DDH due to the narrowness of the birth canal and the forces exerted on the joint capsule, which could favor dislocations [21]; however, this behavior was not evident in this study sample. We consider that, given its relevance in the literature, this variable should be included in future studies with larger sample sizes in order to reassess its significance.

Regarding ethnicity, 44% of the subjects identified as belonging to the Mapuche ethnic group, of whom 41.6% presented DDH. This finding is relevant, as the prevalence of DDH varies according to ethnic group and geographic location [1,14]. This variable should be considered exploratory, as it has not been previously addressed in the literature. Furthermore, the inclusion of these data in a Chilean population allows for comparisons with previously reported findings from other regions and highlights the need for future studies that include areas of the country with a predominance of other ethnic groups, thereby strengthening the current body of evidence. However, no statistically significant association was found between ethnicity and DDH (*p* = 0.862). We consider that this may be attributable to the sample size included in the study, which should be addressed in future research in order to overcome these limitations.

With regard to firstborn status, 37% of the sample were firstborn infants, of whom 33.3% were diagnosed with DDH. No statistically significant relationship was observed between these variables. However, given that the literature recognizes being firstborn as a risk factor, it is considered relevant to maintain this variable in future studies. This may be primarily explained by maternal morphological conditions, as in primiparous women the birth canal tends to be narrower, potentially generating increased mechanical compression on the joint capsule, which could contribute to joint instability, including dislocation or weakening of the articulation.

Regarding feeding type, 52% of the sample received exclusive breastfeeding (EBF), 33% mixed feeding, and 15% exclusive formula feeding. No statistically significant association was found between feeding type and the presence of DDH. Notably, the highest proportion of DDH cases was observed among infants receiving EBF (58.3%). This variable is considered exploratory, as it has not been described in previous studies. It is nonetheless of interest, given that nutrition plays a key role in bone growth and maturation [36]. Therefore, future studies should incorporate information on maternal nutrition during exclusive breastfeeding, as well as analyses of the composition of the infant formulas used.

Of the total sample, 5% had unequal lower limbs (MMII), with no diagnosis of DDH in any of these cases. Similarly, of the 9% who had unequal gluteal folds, only one subject was diagnosed with DDH, with no statistical significance found. In contrast, of the 18% who presented asymmetry in the abduction of the hip joint, 5 subjects were diagnosed with DDH, finding a statistically significant association between these variables (*p* = 0.023). This finding is relevant for the population studied and is consistent with what is reported in the literature [6]. Although the prevalence of these clinical findings varies across studies, the literature does report their presence in DDH. Therefore, these variables should continue to be investigated in this population.

A total of 10% of the sample had a history of breech fetal presentation. When this variable was analyzed in relation to the presence of DDH, only one infant presented both conditions, with no statistically significant association identified. Although breech presentation has been described as a risk factor [21], it was not predominant in our sample. The relevance of this variable lies in the forces exerted on the hips during delivery, particularly when the buttocks present toward the vaginal canal, which may favor joint dislocation. Regarding family history of DDH, 31% of the sample reported prior diagnoses within the family, a condition recognized in the literature as a risk factor [20]. However, only three of the cases diagnosed with DDH presented this antecedent, with no statistically significant association observed between these variables. It is important to note that these findings may be influenced by the sample size, which constitutes a limitation of the study. As this is an exploratory investigation, these variables will continue to be considered in future research, with efforts directed toward including larger sample sizes.

The BLR model demonstrated an accuracy rate of 88%, indicating an adequate predictive capacity to correctly classify the presence or absence of dysplasia. The global significance test of the model suggests that the inclusion of the selected variables significantly improves its explanatory power compared to a model including only the intercept, thereby supporting the usefulness of these predictors. These variables are considered exploratory; therefore, they should be interpreted with caution. Further research is required to validate these findings before establishing a definitive predictive model.

We acknowledge that the Nagelkerke R^2^ value of 0.988 is unusually high and suggests potential overfitting of the model, likely due to the limited number of outcome events (n = 12) relative to the number of predictor variables included. This result should therefore be interpreted with caution. It is important to note that the primary aim of the model was exploratory, seeking to assess the combined contribution of variables such as hip abduction asymmetry and acetabular roof obliquity, both of which demonstrated individual statistical significance (*p* < 0.05).

The descriptive statistics showed that the average values of the acetabular angles were close to 23°, considering that the age of the infants was between 90 and 150 days. This finding could serve as a reference for future studies aimed at establishing normal ranges according to age. It should be noted that the literature establishes an acetabular angle of 30° as the limit above which DDH is diagnosed [2].

Finally, patients diagnosed with DDH exhibited lower mean values in thigh circumference (27.7 cm) and pelvic circumference (39.9 cm), as well as in weight (6388 g) and length (60.3 cm) at the time of examination. These findings suggest that similar or lower values may be associated with a higher likelihood of developing DDH. In this context, the statistically significant association observed between length at the second month and the presence of DDH underscores the importance of considering these variables in future studies with larger sample sizes. These variables are regarded as exploratory in our study and have not been previously reported in the literature. The rationale for this consideration is to explore a potential association between the presence of DDH and infant anthropometry, or a possible relationship between growth—as an indicator of the infant’s nutritional status—and DDH, as well as to evaluate a potential predictive model based on these exploratory variables.

## 5. Conclusions

The present study allowed for the determination of the prevalence of DDH in a sample of the Chilean population, providing relevant information to the international literature, particularly considering that this condition exhibits variability in prevalence according to geographic region. In our sample, a prevalence of 12% was reported, which is high compared to that described by other authors at the regional level. It is important to interpret this prevalence estimate as preliminary and with caution, given the inherent limitations of the study. This finding underscores the need to further investigate population-specific risk factors, thereby motivating the development of future studies in this field.

Likewise, risk factors associated with DDH were identified, highlighting a statistically significant relationship with acetabular roof obliquity, greater involvement of the left hip, and asymmetry in hip abduction.

The BLR model showed an accuracy rate of 88% for the variables acetabular roof obliquity, affected hip, and abduction asymmetry, confirming its usefulness as a predictive variable for DDH. The information obtained provides preliminary bases for the development of clinical studies aimed at generating more locally relevant evidence on this highly relevant pathology in the infant population.

The clinical importance of DDH lies in its high prevalence among pediatric patients [1] and its potential to cause multiple long-term complications, which may even become disabling [3]. Furthermore, this condition is associated with substantial healthcare costs, particularly because its management in adulthood is often surgical [10]. Generating evidence on this condition contributes to clinical decision-making, with a primary focus on the prevention of complications through early diagnosis. Providing robust and context-specific data may help reduce the need for surgical intervention. We consider this study to be an initial step toward advancing the generation of local knowledge on DDH, ultimately aiming to benefit the pediatric population.

As a limitation of the study, it is acknowledged that its observational design does not allow for direct clinical interventions or the establishment of causal relationships. Additionally, selection bias and the limited sample size must be considered. Although the sample was calculated based on the available population, the findings cannot be extrapolated to other regions of the country; therefore, the results should be regarded as exploratory.

In this context, future research should consider alternative methodological designs, larger sample sizes, the inclusion of maternal variables, and the implementation of multicenter studies at the national level.

## Figures and Tables

**Table 1:** Stages of acetabular labral development within the acetabulum.

Cotyloid Eyebrow Development	Frequency
Absence	1
Sparse	27
Moderate	10
Adequate	62

**Table 2:** Descriptive statistics of scalar variables.

Variable	Minimum	Maximum	Mean	Standard Deviation	Confidence Interval-95 CI
Right acetabular angle (°)	14	33.5	23.96	4.15	[23.14; 24.79]
Left acetabular angle (°)	14	34	24.18	4.22	[23.35; 25.02]
Week of gestation	36	42	38.4	1.23	[38.16; 38.64]
Right thigh measurement (cm)	19.5	28	23.21	1.97	[22.81; 23.60]
Left thigh measurement (cm)	19.2	27.9	23.17	1.93	[22.79; 23.55]
Pelvic circumference (cm)	33.7	60.5	40.77	3.40	[40.10; 41.45]
Weight at examination (g)	4830	8720	6616	806.5	[6455.87; 6775.93]
Height at examination (cm)	56.3	67	61.08	2.13	[60.66; 61.50]
Number of children	1	6	2	1	[1.74; 2.14]

## Data Availability

The data presented in this study are available upon request from the corresponding author, as some of these belong to the patient’s medical record, which we are required to protect by law.
